# PDE4 inhibitor mitigates activated CD8+ T cells through NF-κB signaling in Behçet’s syndrome

**DOI:** 10.3389/fimmu.2026.1834685

**Published:** 2026-06-29

**Authors:** Alexandre Le Joncour, Paul Régnier, Anna Maciejewski-Duval, Erwan Charles, Cloé Comarmond, Stéphane Barete, Matheus Vieira, Pierre Fouret, Alexandre Belot, Sébastien Viel, Michelle Rosenzwajg, David Klatzmann, Patrice Cacoub, David Saadoun

**Affiliations:** 1 Sorbonne Université, INSERM, UMR S 959, Immunology-Immunopathology- Immunotherapy (I3); Laboratoire d’excellence TRANSIMMUNOM, RHU iMAP, Paris, France; 2 Biotherapy (CIC-BTi), Hôpital Pitié-Salpêtrière, AP-HP, Paris, France; 3 Department of Internal Medicine and Clinical immunology, Hôpital Pitié-Salpêtrière, AP-HP, Paris, France; 4 AP-HP, Groupe Hospitalier Pitié-Salpêtrière, Unit of Dermatology, DMU3ID, Paris, France; 5 Center of Research on Inflammation, CNRS ERL 8252, Université de Paris, Sorbonne Paris Cite, Laboratoire d’excellence INFLAMEX, Paris, France; 6 Paediatric Rheumatology, Nephrology, Dermatology Unit, National Reference Centre for Rheumatism and Systemic Autoimmune Diseases in Children RAISE, Hospices Civils de Lyon, Lyon, France; 7 Immunology Laboratory, National Reference Centre for Rheumatism and Systemic Autoimmune Diseases in Children RAISE, Hospices Civils de Lyon, Pierre Bénite, France

**Keywords:** apremilast, behçet disease, CD8, NFkappaB, PDE4 inhibition

## Abstract

**Objectives:**

Behçet’s disease (BD) is a systemic vasculitis with inflammatory lesions mediated by cytotoxic T cells and neutrophils. Here, we explore the critical involvement of NF-κB signaling pathway in proinflammatory CD8+ T cells differentiation and disease progress of BD patients.

**Methods:**

We performed microarray gene expression analyses, flow cytometry, immunophenotyping, immunohistochemistry and functional assessments of CD8+ T cells from BD patients and HD.

**Results:**

Transcriptionally, among the 6,595 up-regulated genes in CD8+ T cells of BD vs HD, we highlighted a great enrichment for pathways linked to NF-κB and TLR signaling (i.e. *NFKB1*, *RELB*, *REL*, *TLR1*, *IRF4)*. Phenotypically, CD8+ T cells from BD had a higher expression of phosphorylated NF-κB (pNF-κB, 4.4 ± 0.9 *vs.* 1.8 ± 0.2 in MFI, p = 0.001), were more activated (higher CD11c, CD11b, CD25 and TNF-α, IFN-γ expression) and exhibited more expression of Perforin and Granzyme B (33% ± 9 *vs*. 9 ± 5, p = 0.009, 47% ± 8 *vs.*21% ± 6, p = 0.02, respectively) as compared to HD. Phosphodiesterase-4 (PDE4), an immune cell enzyme that activate the NF-κB pathway was up-regulated in blood and skin lesions of BD. *In vitro* and *in vivo* inhibition of PDE4 strongly inhibited CD8+ T cell activation, cytokine secretion, cytotoxicity and proliferation.

**Conclusion:**

We highlighted that activated CD8+ T cells through NF-κB signaling pathway are instrumental in BD.

## Highlights

- Behçet’s disease patients exhibit NF-κB pathway activation in CD8+ T cells.
- Behçet’s disease CD8+ T cells show heightened activation, cytokine secretion, and cytotoxicity.
- Inhibiting PDE4 mitigates CD8+ T cell activation, cytokine production, and proliferation *in vitro* and *in vivo* and at the transcriptomic level.

## Introduction

Behçet’s disease (BD) is a chronic systemic vasculitis primarily characterized by orogenital ulcerations, but also affecting other organs, including the eye (uveitis), joints (arthritis), skin, brain and vessels (1). This immune-mediated inflammatory disorder involves different vessel types and sizes of the vascular tree and is often complicated by recurrent thrombosis, particularly in the venous compartment. BD may cause several serious morbidities and a fatal outcome. Long-term follow-up study found an overall mortality rate of 5-10% as well as increased standardized mortality ratios among young males. Major causes of death included large vessels and parenchymal central nervous system involvement. The exact pathogenic mechanisms underlying BD are still unclear.

The implication of T cells is supported by pathological studies showing perivascular infiltration of activated CD8+ cytotoxic T cells and polymorphonuclear leucocytes within vasculitic lesions in patients with BD (2). HLA-B51 is by far the most strongly associated genetic factor to BD (3) but others major histocompatibility complex (MHC) class I chain-related (MIC-A and MIC-B) are also susceptibility genes. Increased proportion of activated CD8+ T cells (4, 5) is observed in BD and seems to play an important role in the pathogenesis of this disease. Noteworthy, CD8+ T cells have been shown to be strong inducers of Th1-polarized immune responses (6, 7) in experimental models. These cytotoxic T cells may act against vascular endothelial cells and therefore participate to BD pathophysiology (8). The exact characterization of this population and molecular mechanisms underlying the differentiation of proinflammatory T cells in BD are essentially unknown.

Currently, BD is mainly treated with nonspecific corticosteroids and Disease Modifying Anti-Rheumatic Drugs (DMARDs) which are associated with potential side effects, especially when used in the long-term. To develop more efficient treatments against BD’s persistent inflammation, physicians need a deeper understanding of the disease and its mechanisms. The NF-κB pathway plays a critical role in driving inflammation in several cell types by producing key pro-inflammatory cytokines such as IL-1, IL-6 and TNF-α, which are often elevated in BD (9). Negative regulation of this pro-inflammatory pathway (ie NF-κB pathway) is essential for reducing inflammation and is controlled by the ubiquitination of several protein complexes such as A20 and OTULIN (10). These are proteins that modify these target complexes and their deficiency leads to over-activation of the NF-κB pathway. Haploinsufficiency of A20 is a dominantly inherited, early-onset systemic inflammation that shares with BD many clinical features like recurrent mucosal ulcers, uveitis, arthralgia and thrombosis (11). Few studies have reported NF-κB activation in peripheral blood mononuclear cells (PBMCs) of BD patients (12–14). Phosphodiesterase 4 (PDE4) is an inflammatory cell enzyme which, by degrading the key intracellular signaling messenger cyclic adenosine monophosphate (cAMP), promotes an increase in the production of pro-inflammatory mediators through the NF-κB pathway (15–17). Apremilast is an orally administered small molecule that acts as a PDE4 inhibitor, reducing the activation of the NF-κB pathway. Apremilast showed a therapeutic benefit in treating oral ulcers of BD in phase II and III studies (18, 19) and had recently been approved by the Food and Drug Administration (FDA). However, its biological effects in BD have not yet been established.

Here, we aim to explore the involvement of NF-κB signaling pathway in proinflammatory CD8+T cells in blood and skin lesions and disease activity of BD patients.

## Methods

### Study population

The study population consisted of 50 BD age at diagnosis: 39 (33,45) years), fulfilling the international criteria for BD (20). Clinical characteristics of BD patients are presented in **Table 1**. Twenty-one patients out of them presenting active oral ulcers were treated with Colchicine 1mg/day (n=9) or Apremilast (30mg twice a day) (n=12), for which blood samples were also collected before and after treatment. Blood samples from 34 age- and sex-matched healthy donors (HD), obtained from the Établissement Français du Sang (Hôpital Pitié-Salpêtrière) were used as controls. The study was approved by the Paris VI ethics review board and was performed according to the Helsinki declaration. All patients gave informed consent.

**Table 1:** Main characteristics of patients with Behçet’s disease.

Characteristic	Patients
Age, median year [IQR]	39 (33–45)
Sex, Male n (%)	30 (60)
Geographic origin, n (%)	
Europe	17 (33)
North Africa	21 (40)
Asia	2 (2)
Clinical features, n (%)	
Oral ulcers	47 (93.3)
Genital ulcers	40 (70)
Skin involvement	32 (64)
Ocular involvement	10 (20)
Vascular involvement	9 (18)
Joint involvement	17 (34)
CRP > 10mg/l, n (%)	16 (32)
Medical therapy, n (%)	
Naive	12 (24)
Colchicine alone	13 (26)
Steroids alone (<10mg/d)	13 (26)
Steroids (<10mg/d) + Colchicine	12 (24)

IQR, interquartile Range; CRP, C reactive protein.

### Patient involvement

We provided instructional support to foster knowledge of research concepts and terminology. If the patients asked, we provided them information about the course of the study. Patients will be informed of the publication of the study and an understandable explanation will be given.

### Transcriptomic data of CD8+ T cells

CD8+ T cells were isolated from PBMCs of BD or HD by positive selection using Dynabeads CD8+ isolation kit. Cell purity was ≥ 95%. Total RNA from CD8+ T cells was then extracted using the NucleoSpin RNA kit (Macherey-Nagel) and quantified by NanoDrop ND-1000 spectrophotometer. Samples with RNA concentration < 20 ng/μL were excluded. For quality control, RNA dilution was performed using Agilent RNA 6000 Nano Kit and 1μL of the sample was run on the Nano chip using an Agilent 2100 electrophoresis bioanalyzer. The quality of total RNA was assessed using the electropherogram’s profile and the calculated RNA integrity number (RIN). All samples showed RINs between 7.3 and 9.3. A total of 6 HD CD8+ and 8 BD CD8+ samples were discarded because of their low RNA concentration or low RIN. For Illumina Beadarrays, remaining cRNA samples were prepared using Illumina TotalPre-96 RNA Amp kit (LifeTechnologies) and hybridized to Human HT-12 v4 Beadarrays. Then, raw IDAT files were processed using *illuminaio* R package and concatenated into a single text file. Data were further background-corrected using *limma* R package and interchip batch effects were removed using *ComBat* method from *sva* R package. The following samples numbers remained and were analyzed: 34 CD8+ T cells from HD and 22 CD8+ T cells from BD. Gene and pathway enrichment analyses were performed using *limma* and *GSVA* R packages. More than 5,000 different pathways coming from well-known databases (GeneOntology, PANTHER, KEGG, Reactome and WikiPathways) were used.

### T cells culture and flow cytometry analysis

For *in vitro* experiments, PBMCs from either HD or BD were cultured in CD3/CD28-coated 48 wells plates at 1 million cells/mL using R10 culture medium (Roswell Park Memorial Institute (RPMI) supplemented with BSA, L-glutamine and Penicillin/Streptomycin, all from Gibco) for 5 days, concomitantly with either phosphate-buffered saline (PBS) or Roflumilast (1µM, Sigma Aldrich) or Colchicine (100ng/ml, Thermofisher) as described elsewhere (21). After the culture, samples were antibody-stained following standard protocol with anti-CD3 (1/200), anti-CD8 (1/150), anti-CD69 (1/50), anti-CD4 (1/100), anti-CD11b (1/100) (BioLegend), anti-IFN-γ (1/50), anti-CD11c (1/100) (Miltenyi), anti-TNF-α (1/50), anti-Granzyme B (1/50), anti-Perforin (1/50), anti-CD25(1/100), anti-pNF-κB (1/100) (BD Biosciences) and anti PDE4 (1/50) (Abcam).

### PKA activity

Peripheral blood mononuclear cells (PBMCs) from six patients with Behçet’s disease were cryopreserved prior to analysis. Frozen PBMCs were thawed and cultured in CD3/CD28-coated 48-well plates at a density of 1 × 10^6 cells per well in 1 mL of R10 medium, in the presence or absence of 100 µM Roflumilast. Following culture, cell lysates were prepared according to the manufacturer’s protocol. Protein kinase A (PKA) activity was measured using a colorimetric assay kit (Invitrogen, Ref. EIAPKA) according to the manufacturer’s instructions. Briefly, 40 µL of cell lysate was used for each reaction.

### Immunofluorescence

Paraffin-embedded skin tissue sections from BD pseudo folliculitis or inflammatory skin lesions and control were submitted to sequential indirect immunolabeling with tyramid signal amplification technique using 3-plex composed of DAPI, CD8 and PDE4. The following anti-human antibodies were used: anti-CD8 (ref. MA5-13473, Thermofisher Scientific), anti-PDE4 (ref. ab14628, Abcam).

Before the actual immunolabeling, slides were dewaxed at 72 °C during 30sec before an antigen retrieval step (pH 9, at 95 °C for 20min) was performed. After this, endogenous peroxidases and non-specific binding sites were blocked with hydrogen peroxide (10min at room temperature) and bovine serum albumin solution (10 min at RT, without rinse). Next, primary antibodies were sequentially applied for 45min at 37 °C, then secondary antibodies were incubated for 30min at RT. After the completion of all immunolabeling, spectral DAPI (Akoya Biosciences) was added to the slides for 5min as a counterstaining, and coverslips were mounted with mounting medium. The immunolabeling detection was finally done using Opal^®^ 4-Color Automation IHC kit (Akoya Biosciences). Slides were thereafter scanned with a Axio Scan.Z1 (Zeiss Microscopy) slide scanner, equipped with a Zeiss Colibri 7 LED illuminator and a Hamamatsu ORCA-Flash4.0 CMOS camera, using a PlanApochromat 20x, NA 0.8 objective. The method used to open, process and analyze the subsequent CZI files is described below.

Once the slides were scanned, obtained CZI files were opened using QuPath software in order to extract as much exploitable regions of interest (ROI) as possible, which were directly sent to Fiji version 1.53h software. At this point, using Fiji version 1.53h software, a semi-automated scripted analysis was performed to remove background noise and locally enhance contrast for each channel. Then, nuclei contours (edges) were determined using Difference of Means (DoM) method, once the nuclei contours were identified, their shapes were dilated to take the cell cytoplasm area into account. After that, quantification of the remaining signals inside each determined cell shape was performed. Thereafter, fluorescence, spatial and shape information for each identified cell in each ROI and 3-plex were exported from Fiji, imported to R version 4.1 software and to be easily analyzed with FlowJo version 10.8 software. CD8+ cells, and CD8+ PDE4+ cells were automatically counted and normalized to ROI surface.

### Statistics

Continuous variables are presented with the median and range or with the mean ± SEM. Categorical variables are presented with counts and proportions. Statistical comparisons were performed by using the Mann-Whitney test for quantitative unpaired data, and the Wilcoxon matched pairs test for quantitative paired data. Flow cytometry analyses and *in vitro* experiments were performed blinded to disease status and treatment group. All statistical tests were two-tailed with a significance level of 0.05. Statistical significance was evaluated using GraphPad Prism version 5.00 for Windows (GraphPad Software, San Diego, CA, USA).

## Results

### Molecular signature of CD8+ T cells in BD

**Figure 1:**
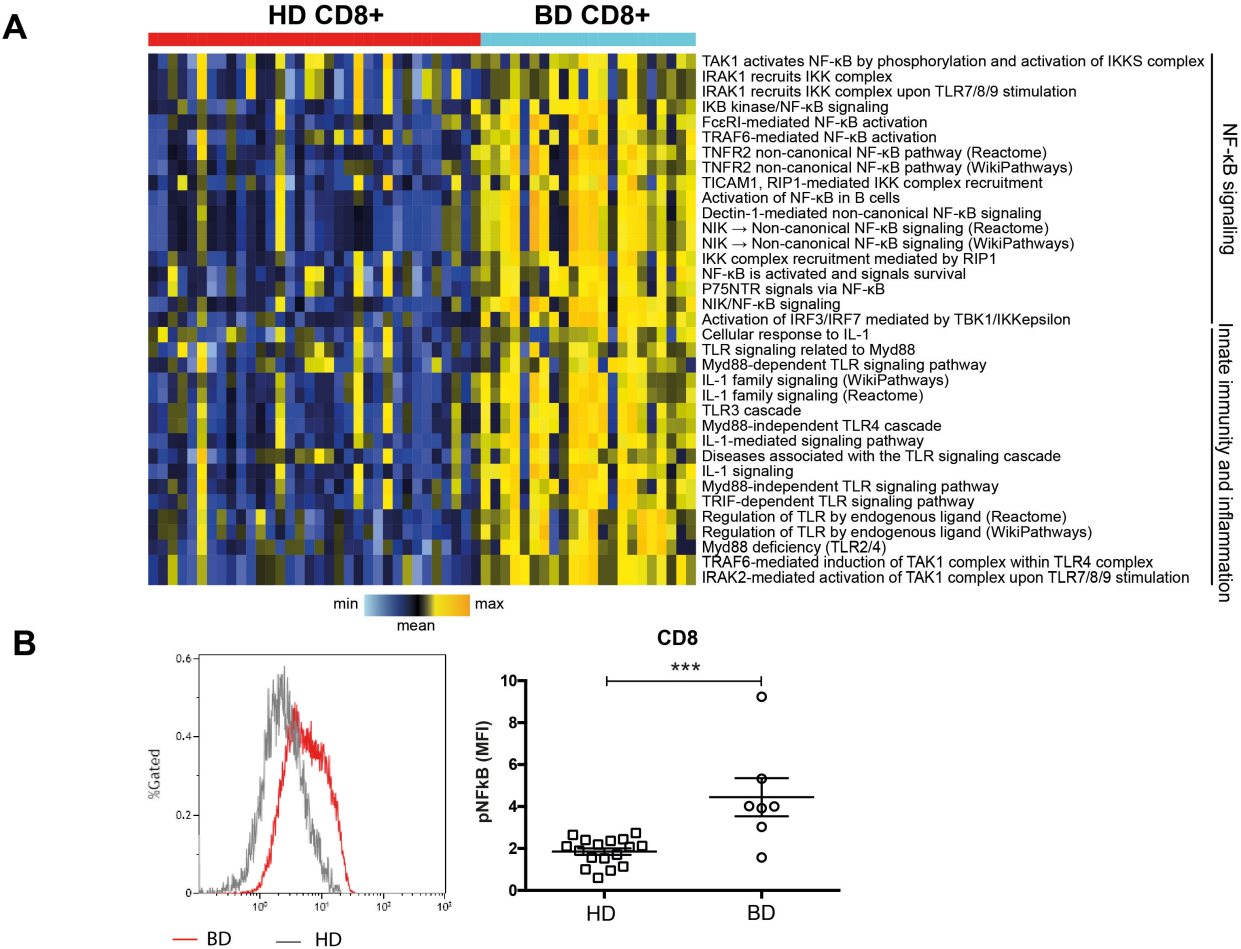
Molecular signature in CD8+ T cells of BD. **(A)** Heatmap showing that NF-κB- and innate immunity/inflammation-related pathways are among the most upregulated functions in CD8+ T cell of BD as compared to HD **(B)** Flow cytometry-based analysis of intracellular pNF-κB in CD8+ T cells from HD and BD. HD, Healthy donors; BD, Behçet’s disease. ***p<0.001.

Transcriptomic differential analysis of CD8+ T cells samples revealed a huge number of dysregulated genes in BD as compared to HD (250 upregulated genes and 260 downregulated genes in BD versus HD with adjusted p-values < 0.01 and Log2FC ≥ 0.5). To better distinguish the biological functions linked to genes, we performed gene sets enrichment analysis between HD and BD. Among the 5,493 tested gene sets, we found 1,254 significantly dysregulated pathways (652 upregulated pathways and 602 downregulated pathways in BD versus HD with adjusted p-values < 0.01 and |Enrichment| ≥ 0.4). Among the top-dysregulated pathways, we noticed a great abundance of NF-κB (such as *IRAK1 recruits IKK complex*, *FcϵRI-mediated NF-κB activation*, *TRAF6-mediated NF-κB activation* and *NIK/NF-κB signaling*)- as well as innate immunity/inflammation-related pathways (such as *TLR signaling related to Myd88*, *TRIF-dependent TLR signaling pathway*, *IRAK2-mediated activation of TAK1 complex upon TLR7/8/9 activation* and *IL-1-mediated signaling pathway*) which were all strongly and significantly up-regulated in BD CD8+ T cells as compared to HD (**Figure 1A**). This was confirmed at the gene level, as among the most strongly upregulated genes in BD compared to HD, we notably found *NFKB1*, *RELB*, *REL*, *TLR1*, *IRF4*, *LY96*, *OASL*, *IFNGR2*, *IFIT2*, *IFTI3* and *IFIH1* genes which are involved in the previously cited biological functions.

We also confirmed the up-regulation of phosphorylated NF-κB (pNF-κB) protein by flow cytometry. As shown in **Figure 1B**, CD8+ T cells from BD had a significantly higher expression of pNF-κB as compared to HD (4.4 ± 0.9 *vs.* 1.8 ± 0.2 in MFI, p = 0.001). Characteristics of patients included in the analysis are shown in **Supplementary Table 1**.

### Marked PDE4 expression by CD8+ T cells in blood and skin lesions of BD

**Figure 2:**
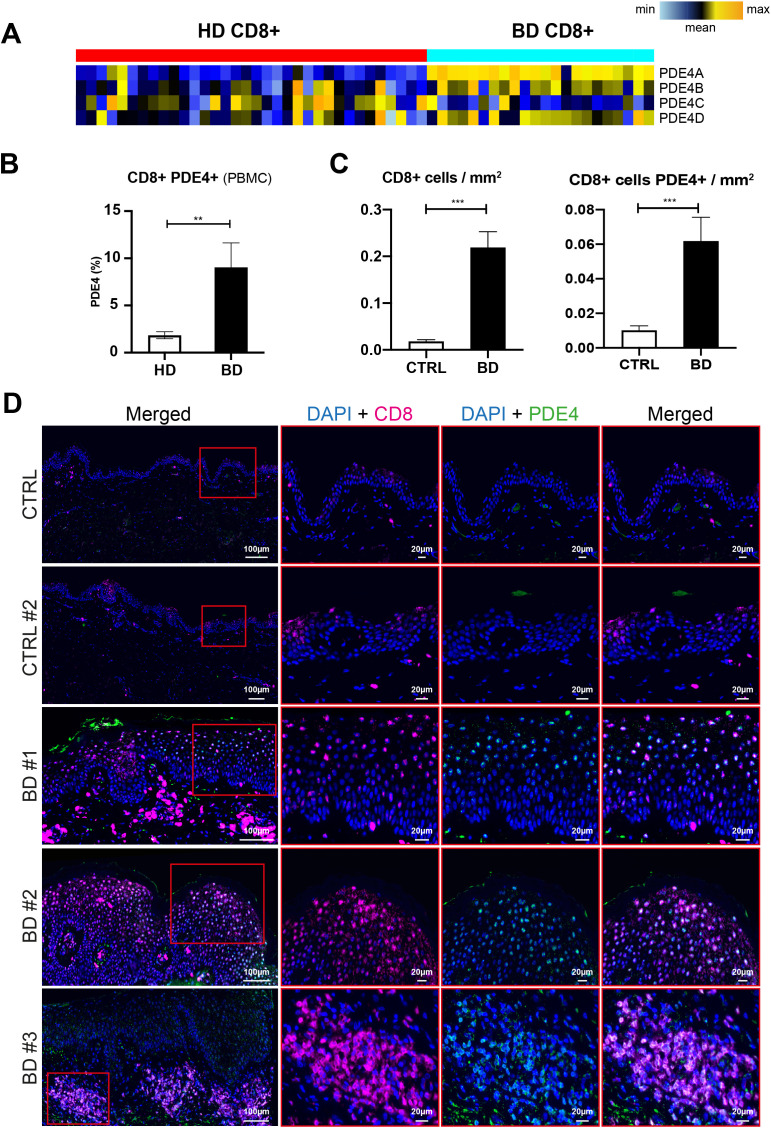
Marked PDE4 expression by CD8+ T cells in blood and skin lesions of BD. **(A)** Heatmap showing PDE4 isoforms genes expression in BD and HD CD8+ T cells. **(B)** PDE4 expression in CD8+ T cells from HD (n=4) and BD (n=9) **(C)** number of CD8+ T cells and CD8+ PDE4+ T cells in BD skin lesion and controls **(D)** Representative images of PDE4 immunostaining in BD skin lesions and control skin slices. *p-value < 0.05, **p-value < 0.01, ***p-value < 0.001. PDE4, phosphodiesterase 4; HD, Healthy donors; BD, Behçet’s disease; CTRL, Control.

PDE4 is an immune enzyme which hydrolyzes the cAMP in 5’-AMP, therefore preventing cAMP to exert its secondary messenger function. This enzyme is available in 4 isoforms, each of them being coded by a single gene: *PDE4A, PDE4B, PDE4C* and *PDE4*D. Our transcriptomic analyses revealed a significantly higher expression of 3 out of 4 PDE4 isoforms mRNAs [*PDE4A* (adjusted p-value ≈ 4e-22), *PDE4B* (adjusted p-value ≈ 4.8e-6) and *PDE4D* (adjusted p-value ≈ 2.6e-4)] in BD CD8+ T cells as compared to HD (**Figure 2A**). We confirmed these results at the protein level by flow cytometric analysis of PDE4 in CD8+ T cells from BD and HD (9% ± 2.6 vs 1.8% ± 0.4, p=0.003) (**Figure 2B**). To confirm and illustrate the overexpression of PDE4 genes in BD CD8+ T cells at the protein level, we analyzed by immunofluorescence BD skin lesions and normal skin slices. Density of CD8+ T cells was increased in BD as compared to control skin (0.018 ± 0.003/mm^2^ vs 0.22± 0.03/mm^2^, p < 0.0001). Moreover, number of CD8+ PDE4 + T cells was also increased in BD skin lesions as compared to control skin (0.010 ± 0.003/mm^2^ vs 0.062 ± 0.01/mm^2^, p=0.0001) (**Figure 2C**). Representative images are shown in **Figure 2D**.

### PDE4 inhibitors modulates NF-κB pathway activation in BD

**Figure 3:**
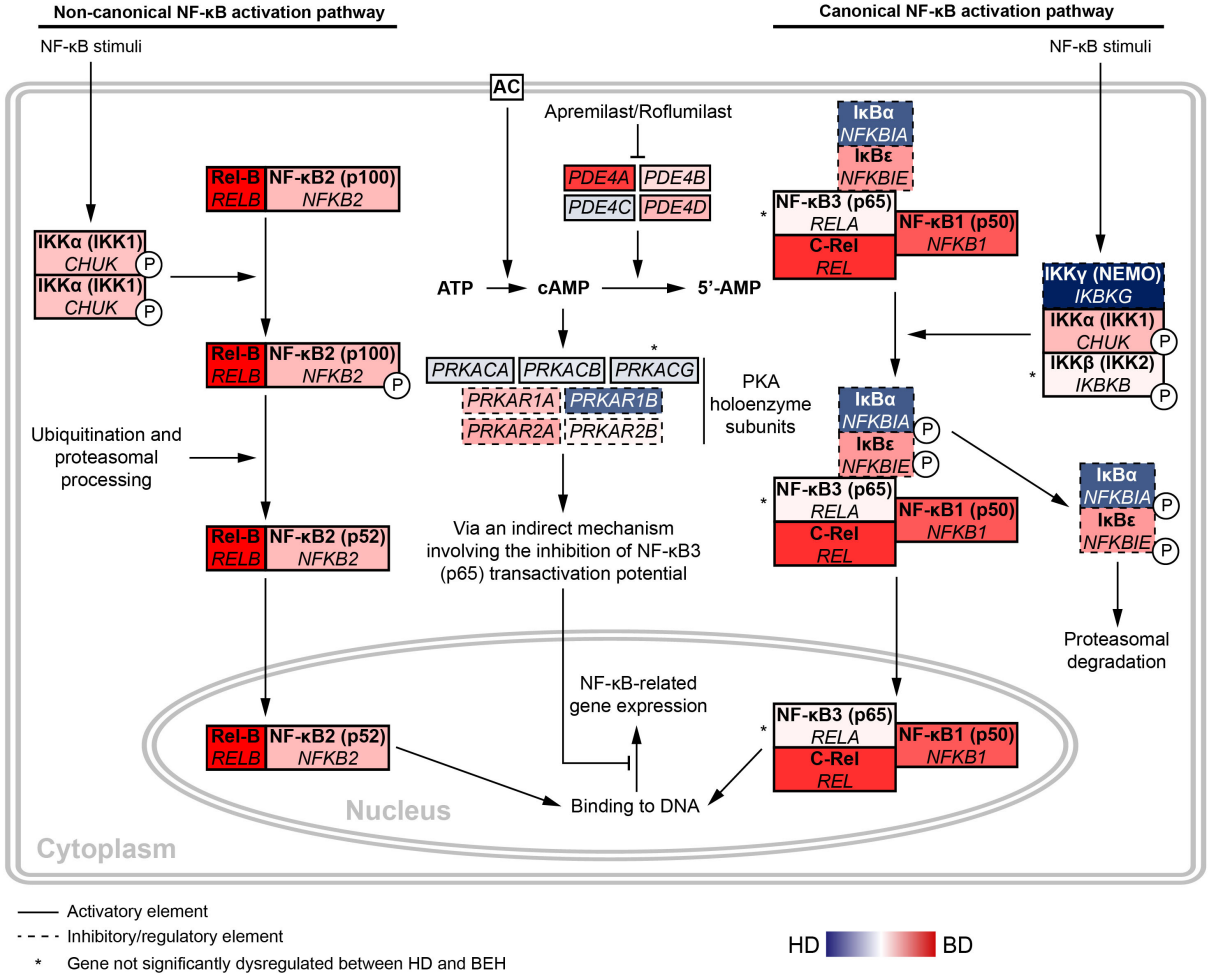
PDE4/Apremilast axis modulates NF-κB pathway activation in BD. Representative network of all the genes contained in the pathways presented in the heatmap using the STRING interaction database and found overactivated in BD CD8+ T cells as compared to HD. White-to-red colors indicate up-regulation of the gene in BD CD8+ T cells, whereas blue-to-white colors indicate up-regulation of the gene in HD CD8+ T cells. The contour of each box is colored according to the role of the protein in the signaling cascade: plain lines indicate activator elements, whereas dashed lines indicate inhibitory elements. HD, Healthy donors; BD, Behçet’s disease.

In order to synthesize our previous results, we reproduced on the **Figure 3** the global NF-κB protein signaling pathway including its canonical and non-canonical activation pathways. To ease the understanding of these interactions, we colored each box according to the strength of the up- or down-regulation of the associated genes (using the log-fold change) obtained in our transcriptomic dataset. White-to-red colors indicate up-regulation in BD CD8+ T cells, whereas white-to-blue colors indicate up-regulation in HD CD8+ T cells. We also adapted the contour of each box according to the role of the protein in the signaling cascade: plain lines indicate activator elements, whereas dashed lines indicate inhibitory elements. Our graph shows that almost all genes coding for activator elements of the NF-κB cascade, both regarding the canonical and non-canonical activation pathways, are significantly upregulated in BD CD8+ T cells as compared to HD, such as *CHUK*, *REL*B, *NFKB2*, *REL* and *NFKB*1. *RELA* and *IKBKB* genes are also upregulated in BD, but the adjusted p-values do not reach significance. On the contrary, almost all genes coding for inhibitory elements of the NF-κB cascade, especially regarding the canonical activation pathway, are significantly downregulated in BD CD8+ T cells as compared to HD, such as *NFKBIA* and *IKBKG*. The only exception is for the gene *NFKBIE* (coding for an inhibitory element of the NF-κB cascade) which is significantly upregulated in BD CD8+ T cells as compared to HD.

We also represented in **Figure 3** connections between NF-κB, PDE4 and cAMP intracellular molecules. cAMP, when not degraded by PDE4, can activate other intracellular actors, such as PKA holoenzymes, ultimately leading to NF-κB pathway inhibition (22). We previously showed that 3 out of 4 PDE4 subunits were significantly upregulated in BD CD8+ T cells as compared to HD (*PDE4A*, *PDE4B* and *PDE4D*), which suggests an increased activity of PDE4 enzyme in BD. So, the supposed subsequent strong decrease of intracellular cAMP in BD would lead to a decreased PKA activity, therefore dampening the inhibition exerted by PKA on NF-κB-related gene expression. Furthermore, 2 out of 3 genes composing the activator subunits of PKA holoenzyme are significantly down-regulated in BD CD8+ T cells as compared to HD (*PRKACA* and *PRKACB*), and the last one (*PRKACG*) is also down-regulated. Additionally, 3 out of 4 inhibitory subunits of PKA holoenzyme are significantly upregulated in BD CD8+ T cells as compared to HD (*PRKAR1A*, *PRKAR2A* and *PRKAR2B*), whereas only 1 of these inhibitory subunits is significantly downregulated in BD CD8+ T cells as compared to HD. These results also suggest that PKA activity is decreased in BD as compared to HD, which could, at least partially, contribute to the great NF-κB signaling cascade up-regulation in this disease.

Together, these data strongly suggest that targeting PDE4 using medications like Apremilast or Roflumilast could help to dampen the NF-κB overexpression seen in BD. In fact, the PDE4 inhibition would lead to increased intracellular cAMP, therefore potentially increasing PKA activity simply by enzymatic affinity, which would ultimately lead to a more potent inhibition of the NF-κB signaling by the PKA.

### PDE4 inhibitors (i.e. Roflumilast) decrease *in vitro* CD8+ T cells activation, cytotoxicity and proliferation

**Figure 4:**
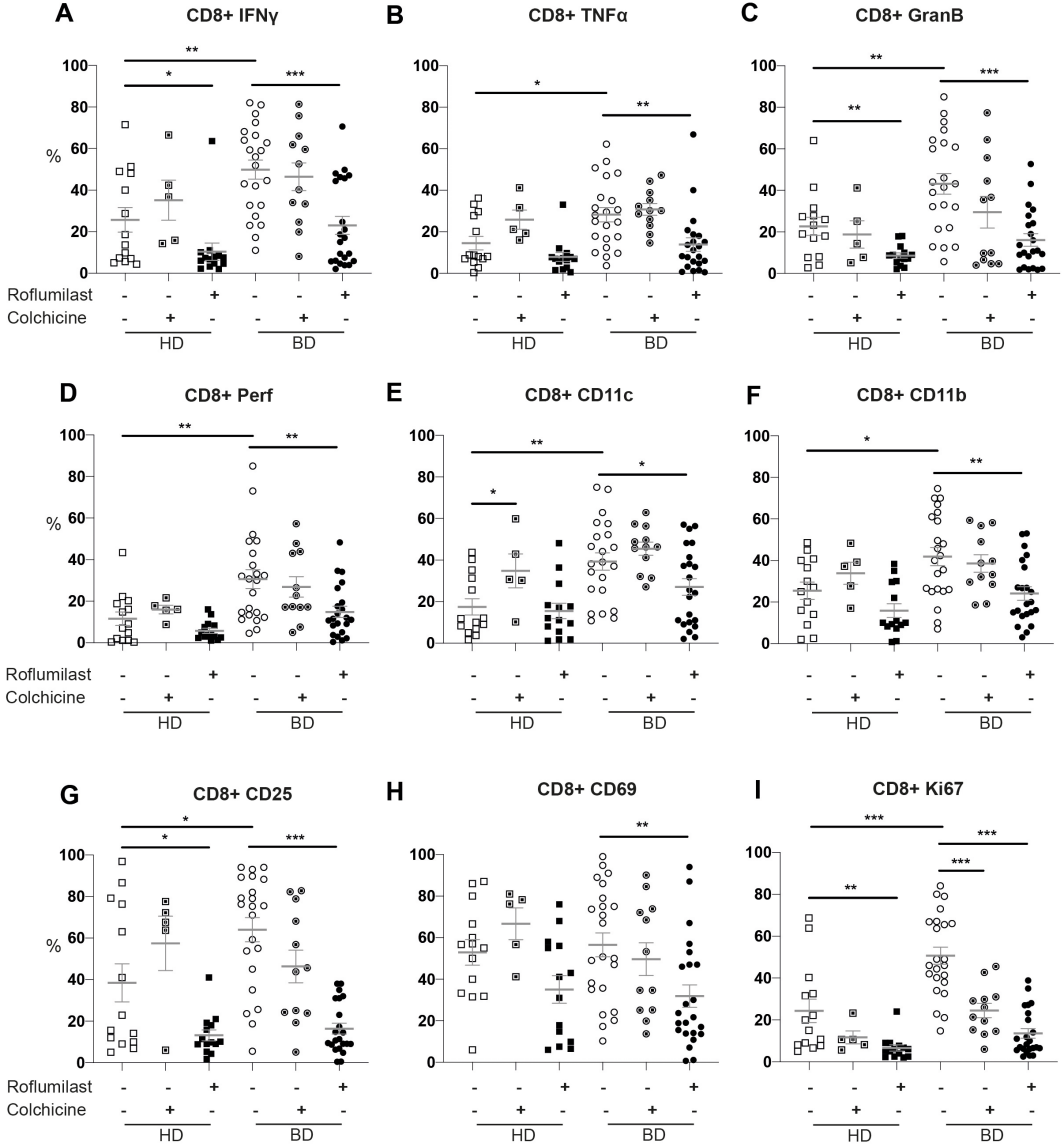
Effect of PDE4 inhibition on activation, cytotoxicity and proliferation in CD8+ T cells of BD. PBMCs from HD and BD were cultured for 5 days with or without Roflumilast (HD: n= 14, BD: n= 22) (1µM) or Colchicine (HD: n= 5, BD: n= 12) (100ng/mL) **(A–D)** Intracellular (IFN-γ, TNF-α, Perforin and Granzyme B) and **(E–H)** surface (CD11c, CD11b, CD25 and CD69) markers were antibody-stained and analyzed by flow cytometry. **(I)** The proliferation marker Ki-67 was analyzed by flow cytometry after intranuclear staining. Data are given as mean ± SEM of > 3 different experiments. For comparisons of untreated versus treated groups, Wilcoxon matched pairs test were used. For comparisons of HD versus BD groups, Mann-Whitney tests were used. *p-value < 0.05, **p-value < 0.01, ***p-value < 0.001. HD, Healthy donors; BD, Behçet’s disease.

To further analyze the biological effect of PDE4/Apremilast axis, we then aimed to study by flow cytometry the *in vitro* effect of Roflumilast (i.e., a PDE4 inhibitor) on CD8+ T cells activation markers in BD and HD. Colchicine was used as a control. First, we showed that BD CD8+ T cells are more activated and present higher expression of cytotoxicity markers as compared to HD. Intracellular IFN-γ, TNF-α, Perforin and Granzyme B (**Figures 4A–D**) were increased in BD as compared to HD (50% ± 5 *vs.* 26% ± 6, p = 0.0026, 28% ± 3 *vs.* 14% ± 3, p = 0.01, 31% ± 5 *vs*. 11% ± 3, p = 0.001, 43% ± 5 *vs.*23% ± 4, p = 0.007, respectively). Surface markers, such as CD11c, CD11b, CD25 were also increased in BD as compared to HD (**Figures 4E–H**) (39% ± 4 *vs.* 17% ± 4, p < 0.001, 42% ± 4 *vs.* 25% ± 4, p = 0.01, 64% ± 6 *vs.* 38% ± 9, p =0.02 and 57% ± 6 *vs.* 53% ± 6, p = 0.7, respectively). Furthermore, BD CD8+ T cells also showed increase Ki-67 expression as compared to HD (51% ± 4 vs. 24% ± 6, p < 0.001) (**Figure 4I**).

Secondly, Roflumilast treatment induced a marked reduction of intracellular markers IFN-γ, TNF-α, Perforin and Granzyme B (**Figures 4A–D**) in BD CD8+ T cells as compared to untreated BD controls (50% ± 5 *vs.* 23% ± 4, p < 0.001, 28% ± 3 *vs.* 14% ± 3, p = 0.004, 31% ± 5 *vs.* 15 ± 3, p = 0.004, 43% ± 5 *vs.* 16% ± 3, p < 0.001, respectively). Roflumilast treatment also induced a marked reduction of CD11c, CD11b, CD25 and CD69 activation markers in BD CD8+ T cells as compared to untreated BD controls (**Figures 4E–H**) (39% ± 4 *vs.* 27% ± 4, p = 0.04, 42% ± 4 *vs.* 24% ± 3, p = 0.002, 64% ± 6 *vs.* 16% ± 3, p <0.001 and 57% ± 6 *vs.* 32% ± 5, p = 0.003, respectively). Roflumilast treatment also significantly abrogated proliferation of BD CD8+ T cells as shown by Ki-67 staining (51% ± 6 vs. 14% ± 2, p < 0.001) as well as in HD CD8+ T cells (24% ± 6 vs. 7%± 1, p= 0.005) (**Figure 4I**). Roflumilast had no effect on TNF-α, Perforin, CD11c, CD11b and CD69 markers but induced a slight reduction of IFN-γ (26% ± 6 *vs.* 10% ± 4, p = 0.04), Granzyme B (23% ± 4 *vs*. 9% ± 1, p=0.05) and CD25 (38% ± 9 *vs.* 13% ± 3, p = 0.01) in HD CD8+ T cells (**Figure 4**). Colchicine had no effect on intracellular markers (IFN-γ, TNF-α, Perforin and Granzyme B) nor on surface markers (CD11c, CD11b, CD25 and CD69). Representative images of significant flow cytometry analyses are shown in **Supplementary Figure 1**.

Lastly, Roflumilast treatment induced a reduction of pNF-κB expression in BD CD8+ T cells (2.9 ± 0.24 vs. 2.5 ± 0.25in MFI, p = 0.003) whereas it only had a little effect in HD CD8+ T cells (1,8 ± 0.3 vs. 1,7 ± 0.3 in MFI, p = 0.1).

Lastly, given the known role of PDE4 inhibition in increasing intracellular cAMP levels and downstream PKA signaling, we additionally assessed PKA activity in PBMCs from BD patients cultured for 3 days in the presence or absence of roflumilast. PKA activity significantly increased after culture with roflumilast compared with untreated conditions [median 0.189 (IQR 0.180–0.192) vs 0.361 (IQR 0.326–0.503), respectively; paired Wilcoxon test, p=0.03].

Together, these results demonstrate that *in vitro* inhibition of PDE4 *via* Roflumilast dampens the activation and proliferation profiles of CD8+ T cells in BD.

### PDE4 inhibitors (i.e. Apremilast) inhibits *in vivo* CD8+ T cells activation, cytotoxicity and proliferation

**Figure 5:**
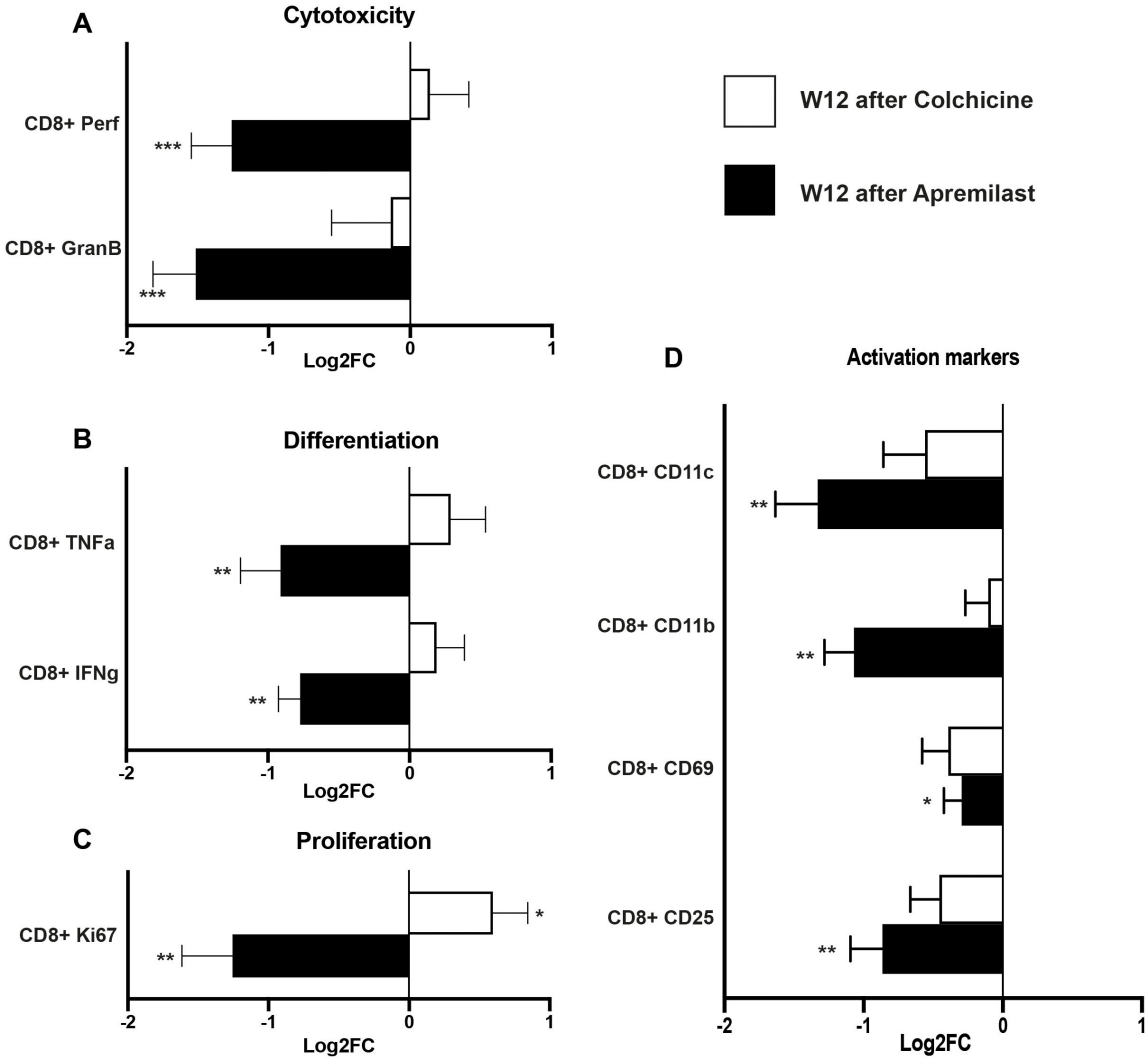
Apremilast inhibits *in vivo* CD8+ T cells activation, cytotoxicity and proliferation in BD. CD8+ T cells from BD treated with Apremilast (n = 12) or Colchicine (n = 9) were analyzed at Week 0 (W0) and Week 12 (W12) by flow cytometry for **(A, B)** intracellular (IFN-γ, TNF-α, Perforin and Granzyme B), surface (CD11c, CD11b; CD25 and CD69) markers **(D)** and **(C)** proliferation. For Colchicine and Apremilast, we independently computed the Log2FC between W12 and W0 results for each displayed feature. A Log2FC < 0 indicates that the treatment induces at W12 a decrease of the feature percentage as compared to the associated W0 baseline control. The opposite applies when Log2FC > 0. Data are given as mean ± SEM of > 3 different experiments. Wilcoxon matched pairs tests were used. *p-value < 0.05, **p-value < 0.01, ***p-value < 0.001. HD, Healthy donors; BD, Behçet’s disease.

To further address the impact of PDE4 inhibition on CD8+ T cells in BD, we studied the *in vivo* effect of Apremilast in BD patients. To do so, we treated BD patients with Apremilast, an orally administered inhibitor of PDE4 (30mg twice a day) and analyzed CD8+ T cells by flow cytometry at baseline (W0) and 12 weeks (W12) after treatment. We also analyzed, as a control, patients treated with Colchicine before and after 12 weeks of treatment. We computed the logarithmic base 2 fold change (Log2FC) between W12 conditions and their W0 counterparts, for Apremilast and Colchicine separately. All patients clinically improved after treatment Behçet’s Disease Current Activity Form (BDCAF) scores significantly decreased after apremilast treatment [median 2 (IQR 2–2) vs 1 (IQR 0–1), Wilcoxon signed-rank test, p=0.001]. As shown in **Figure 5**, we observed a significant reduction of the cytotoxic markers Perforin and Granzyme B (Log2FC: -1,3 ± 0.3, p < 0.001, -1.5 ± 0.3, p = 0.001, respectively) as compared to W0 (**Figure 5A**) but also of IFN-γ and TNF-α cytokines secretion after 12 weeks of Apremilast (Log2FC: -0.8 ± 0.15, p = 0.001, -0.9 ± 0.3, p = 0.003) (**Figure 5B**). Moreover, Apremilast treatment significantly reduced *in vivo* CD8+ T cells activation markers such as CD11c, CD11b, CD25 and CD69 (Log2FC: -1.3 ± 0.3, p = 0.002, -1.06 ± 0.2, p = 0.004, -0.09 ± 0.2, p = 0.003 and -0.3 ± 0.1, p = 0.02, respectively) as compared to W0 (**Figure 5D**) and also decreased proliferation of these cells (Log2FC: -1.3 ± 0.4 *vs*. 24% ± 8, p = 0.008) (**Figure 5C**). However, Colchicine did not decrease cytotoxic markers, IFN-γ and TNF-α cytokines secretion, nor CD8+ T cells activation or proliferation markers (**Figures 5A–D**). Representative images of significant flow cytometry analyses are shown in **Supplementary Figure 2**.

Together, our results clearly demonstrated here that *in vivo* inhibition of PDE4 with Apremilast greatly decreases CD8+ T cells activation, proliferation and cytotoxicity in BD.

## Discussion

The NF-κB pathway is tightly regulated through multiple post-translational mechanisms. Ubiquitination of intermediate protein complexes induces the NF-κB pathway. However, deubiquitinases such as A20, OTULIN and others function as negative regulators of NF-κB signaling. Thus, loss-of-function mutations of A20 drastically induces the NF-κB pathway and have recently been described in humans in a phenotype very close to BD (23). The stimulation of cells from patients with HA20 leads to increased NF-κB activation associated with increased production of multiple pro-inflammatory cytokines (24) which is clinically relevant as no single treatment strategy is effective for all patients. NF-kB dysregulation also included mutations in RELA (encoding p65, named RELA haploinsufficiency) recently shown to produce an autoinflammatory condition, that resembles HA20 in many ways (25). The NF-κB pathway could thus represents an interesting lead in the pathophysiology of BD but has not yet been widely studied. Hamzaoui et al. and Verrou et al. previously found an increased signaling of NF-κB in PBMCs of BD patients (13, 26). Djeraba et al. reported the preferential nucleus translocation of NF-κB in PBMCs of BD which can be inhibited with all-trans-retinoic acid (ATRA) (12). In the present study, we identified by transcriptomic analysis a marked upregulation of both canonical and alternative NF-κB pathway in CD8+ T cells of BD as compared to HD. Transcriptomic analyses allowed us to underline that almost all activator and inhibitory elements involved in the NF-κB pathway were, respectively, up- and down-regulated in BD as compared to HD. NFkB target genes are involved in apoptosis, cell proliferation, JAK/STAT pathway activation, cellular interaction and a positive NFkB feedback loop. Targeting this complex pathway remains challenging.

Phosphodiesterase 4 (PDE4) is an immune and inflammatory cell enzyme which, by degrading the key intracellular signaling messenger cAMP, promotes the NF-κB pathway activation (15–17). PDE4 inhibition alleviates several proinflammatory cytokines/chemokines in various cell types both *in vitro* and *in vivo* includin*g* TNF-α in stimulated PBMCs/monocytes and NK cells (15). Apremilast is an orally administered small-molecule that acts as an inhibitor of PDE4 and which showed a therapeutic benefit in treating oral ulcers of BD in phase II and III studies (18, 19). Here, we pointed out the marked upregulation of PDE4 levels in circulating CD8+ T cells at the transcriptomic level as well as *in vivo* and in skin lesion of BD patients. Functionally, BD CD8+ T cells exhibited increased cell surface activation markers, secretion of IFN-γ and TNF-α, proliferation and cytotoxic features (Perforin and Granzyme B) as compared to HD.

We highlighted that *in vitro* (with Roflumilast) but also *in vivo* (with Apremilast) inhibition of PDE4 alleviates CD8+ T cells activation by dampening NF-κB signaling pathway. In other conditions (such as psoriasis, rheumatoid arthritis), Apremilast inhibits several pro-inflammatory cytokines/chemokines in various cell types both *in vitro* and *in vivo*, including TNF-α in stimulated PBMCs/monocytes and NK cells (15). Specific PDE4 inhibition in CD8+ T cells also showed a decreased secretion of IFN-γ by CD8+ T cells in chronic obstructive pulmonary disease patients (27). Altogether, we demonstrated that activated CD8+ T cells through PDE4/NF-κB signaling axis is instrumental in the pathophysiology of BD and added key mechanisms of cellular inflammation in BD to current knowledge.

We pointed out a possible mechanism behind beneficial clinical effects of Apremilast in BD. We focused on the specific effect of PDE4 inhibitors on PDE4/NF-κB axis in CD8+ T cells. However, as both NF-κB and PDE4 are ubiquitously expressed in many types of cells, we could hypothesized that Apremilast may act more widely. It could for instance additionally inhibits NF-κB signaling and its deleterious effects in neutrophils, monocytes/macrophages and endothelial cells. Indeed Wu et al. have shown that NF-κB was activated in BD macrophages (28), and there is also evidence for NF-κB activation in BD neutrophils (29). This point opens a new avenue in the research of BD and deserves further studies.

In conclusion, our findings highlighted that CD8+ T cells are highly activated in BD and strongly upregulated NF-κB signaling pathways in blood and inflammatory lesions. These results further underline the key role of CD8+ T cells and the NF-κB signaling pathway in BD pathophysiology. Targeting NF-κB signaling by PDE4 inhibition abrogates CD8+ T cells activation and represent a key therapeutic option in BD.

## Data Availability

The datasets presented in this study can be found in online repositories. The names of the repository/repositories and accession number(s) can be found below: E-MTAB-17120, https://www.ebi.ac.uk/biostudies/arrayexpress/studies/E-MTAB-17120.

## Supplementary material

Supplementary Figure 1 Representative flow cytometry analysis of **Figure 4**.

Supplementary Figure 2 representative flow cytometry analysis of **Figure 5**.
