# Supplementary figures and images for: PDE4 inhibitor mitigates activated CD8+ T cells through NF-κB signaling in Behçet’s syndrome
Source: Front Immunol. 2026 Jun 29;17:1834685. doi: 10.3389/fimmu.2026.1834685 (PMC13359495; doi:10.3389/fimmu.2026.1834685)

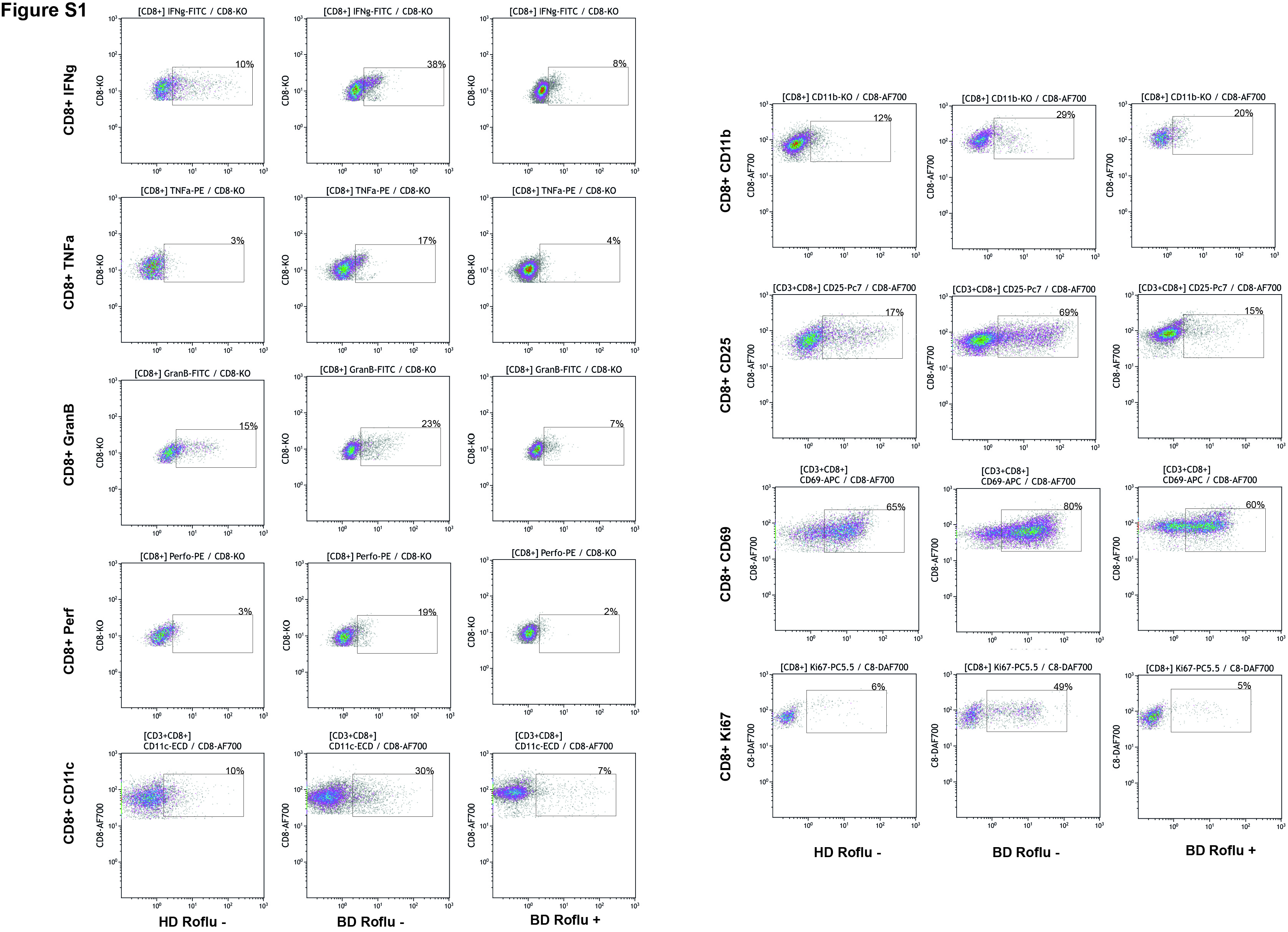

Supplement: Supplementary Figure 1 — Representative flow cytometry analysis of Figure 4. [file Image1.jpeg]

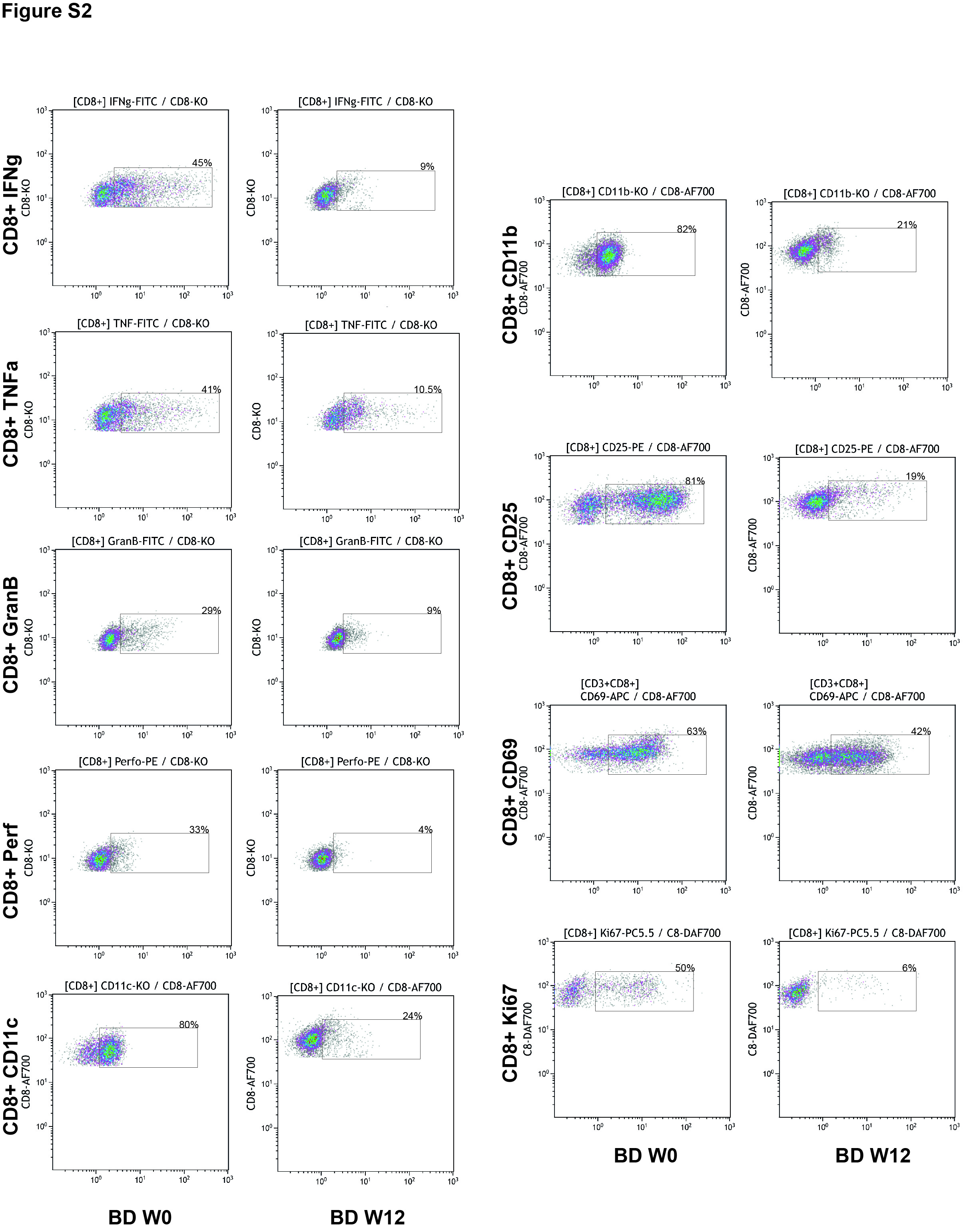

Supplement: Supplementary Figure 2 — representative flow cytometry analysis of Figure 5. [file Image2.jpeg]
