# Supplementary material for: PDE4 inhibitor mitigates activated CD8+ T cells through NF-κB signaling in Behçet’s syndrome
Source: Front Immunol. 2026 Jun 29;17:1834685. doi: 10.3389/fimmu.2026.1834685 (PMC13359495; doi:10.3389/fimmu.2026.1834685)
Supplement: Supplementary file 3 [file Table1.docx]

| **Table S1 : Main Characteristics of patients with Behçet’s disease involved in the transcriptomic analysis** | |
| --- | --- |
|  | all |
|  | N=22 |
| Age, median year [IQR] | 30 [27-40] |
| Sex, Male n (%) | 17 (77) |
|  |  |
| **Geographic origin, n (%)** |  |
| Europe | 10 (45) |
| North Africa | 12 (55) |
|  |  |
| **Clinical features, n (%)** |  |
| Oral ulcers | 22 (100) |
| Genital ulcers | 15 (68) |
| Skin involvement | 13 (59) |
| Ocular involvement | 8 (36) |
| Vascular involvement | 5 (23) |
| Joint involvement | 10 (45) |
|  |  |
| CRP > 10mg/l**, n (%)** | 10 (45) |
|  |  |
| **Medical therapy, n (%)** |  |
| Naive | 5 (23) |
| Colchicine alone | 13 (59) |
| Steroids (<10mg/d) + Colchicine | 4 (18) |
| *IQR: interquartile Range, CRP: C reactive protein* | |
